# Changing QRS Morphology: What is the mechanism?

**Published:** 2006-01-01

**Authors:** Majid Haghjoo, Arash Arya, Mohammad Reza Dehghani, Mohammad Ali Sadr-Ameli

**Affiliations:** Department of Pacemaker and Electrophysiology, Rajaie Cardiovascular Medical and Research Center, School of Medicine, Iran University of Medical Sciences, Tehran, Iran

ECG in sinus rhythm with ventricular preexcitation and changing QRS morphology was seen that was initially interpreted  as the multiple accessory pathway from elsewhere. (Figure 1A).

The following mechanisms are potentially involved in the electrogenesis of changing QRS morphology in WPW syndrome: 1) multiple accessory pathways [1]; 2) simultaneous occurrence of aberrant atrioventricular conduction with accessory pathway conduction [2]; 3) ventricular fusion of preexcited sinus impulse with ectopic impulse.

Electrophysiologic study showed short PR (75 ms) interval with wide QRS (152 ms) and negative HV (-12 ms) interval. No change in delta wave polarity was observed during HRA and CS pacing. In full preexcitation, no breakthrough was seen in the CS. During incremental ventricular pacing, atrial breakthrough site is initially recorded on the HRA catheter and then changed to distal pole of CS catheter with progressive decrease in pacing cycle length. During ventricular pacing at cycle length of 500 ms (S1), earliest atrial activity is recorded on HRA catheter.

Changing QRS could not be explained by presence of *multiple APs* because only right-sided AP had bidirectional conduction and no distal CS breakthrough was seen simultaneous with changing QRS morphology. The possibility of *aberrant conduction* is excluded by presence of negative HV interval in the beats with differing QRS morphology. No sinus cycle length variation before and after the beats with different morphologies are against the occurrence of functional LBBB. The prematurity of ventricular electrogram in His recording catheter with variable HV (H-electrogram is recorded after V-electrogram in second beat and before V-electrogram in third beat) and fixed V-RB intervals (interval from ventricular electrogram in His to the RB potential) are compatible with *ventricular fusion of preexcited sinus impulse with ectopic ventricular impulse* originating from parahissian area (explaining LBBB and inferior axis morphology of the beats with changing QRS) but not from the His bundle or RBB itself  (because H-electrogram and RB potential is recorded after V-electrogram in the second beat with greater degree of ventricular fusion)(Figure 1B).

## Figures and Tables

**Figure 1:**
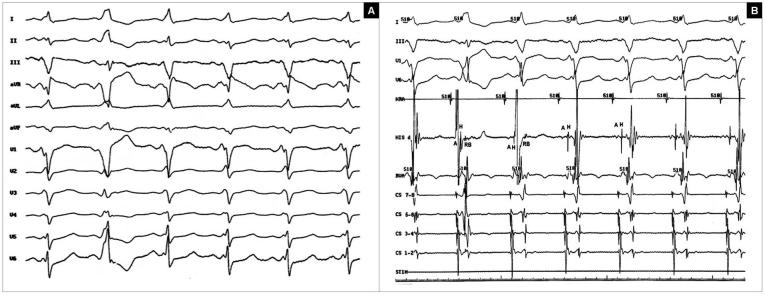
**A** This electrocardiographic tracing shows sinus rhythm with ventricular preexcitation and changing QRS morphology in the second and third beats of the tracing. **B** Intracardiac electrograms and surface ECG recording during the variation of the QRS complexes. The second and third complexes are beats with different degrees of fusion. Note that A-A, H-H, V-V (RVA), and AH intervals are the same in all beats. The main difference of fusion beats with preexcited sinus beats is the variation of HV interval in fusion beats with fixed V-RB interval, indicating the retrograde invasion of the RBB by ectopic impulse arising form the low RVOT area. **HRA**=high right atrium; **His**=his bundle; **RVA**=right ventricular apex; **CS**=coronary sinus; **RVOT**=right ventricular outflow tract; **RBB**=right bundle branch
